# Effect of leukoaraiosis on collateral circulation in acute ischemic stroke treated with endovascular therapy: a meta-analysis

**DOI:** 10.1186/s12883-023-03266-8

**Published:** 2023-06-01

**Authors:** Wang Chen, Yijie Qin, Shuna Yang, Lei Yang, Yutong Hou, Wenli Hu

**Affiliations:** 1 Department of Neurology, Beijing Chaoyang Hospital, Capital Medical University, 8 Gongti South Road, Chaoyang, Beijing, 100020 China; 2 Department of Emergency, Rizhao People’s Hospital, Rizhao, Shandong China

**Keywords:** Cerebral small vessel disease, Leukoaraiosis, Large vessel occlusion, Collateral circulation, Endovascular therapy, Meta-analysis

## Abstract

**Background and objective:**

The recruitment of collateral circulation correlates with a balance of the microvasculature. Uncertainty remains to be made about the association of leukoaraiosis with leptomeningeal collaterals. To explore the effect of leukoaraiosis on leptomeningeal collaterals in patients treated with endovascular therapy.

**Methods:**

Observational studies exploring the correlation between leukoaraiosis and leptomeningeal collaterals in large vessel occlusion treated with endovascular therapy were searched from PubMed, EMBASE, and Cochrane Libraries databases. Two independent reviewers retrieved eligible literature, extracted purpose-related data, and utilized the Newcastle–Ottawa Scale to evaluate the risk of bias. A Mantel–Haenszel method was used to calculate the odds ratio (OR). Meta-regression and subgroup analyses were conducted to clarify heterogeneity.

**Results:**

Data from 10 studies with 1606 patients were extracted for pooled analysis. Compared to non-severe leukoaraiosis, patients with severe leukoaraiosis showed significant relevance to poor leptomeningeal collaterals (OR, 2.13; 95% confidence interval [1.27–3.57]; *P* = 0.004). Meta-regression indicated that sample size (coefficient = -0.007299, *P* = 0.035) and the number of female patients (coefficient = -0.0174709, *P* = 0.020) were sources of heterogeneity. Furthermore, all of the countries (USA versus France versus China, Q = 3.67, *P* = 0.159), various assessment scales of leukoaraiosis (the Fazekas scale versus Non-Fazekas scales, Q = 0.77, *P* = 0.379), and different imaging methods of leukoaraiosis (computed tomography versus magnetic resonance imaging, Q = 2.12, *P* = 0.146) and leptomeningeal collaterals (computed tomography angiography versus digital subtraction angiography, Q = 1.21, *P* = 0.271) showed no contribution to the effect size.

**Conclusion:**

Severe leukoaraiosis is associated with poor leptomeningeal collaterals in patients treated with endovascular therapy. Further studies may focus on whether the finding applies to different stroke subtypes.

**Supplementary Information:**

The online version contains supplementary material available at 10.1186/s12883-023-03266-8.

## Introduction

Among patients with large vessel occlusion in the anterior circulation, endovascular therapy (EVT) has been demonstrated as the first-line therapy [1]. These patients who benefit from EVT reflect a small irreversible infarction (core infarction) and extensive rescue ischemia (penumbra) of brain tissue on preoperative imaging evaluation [2]. A good leptomeningeal collateral, supplying abundant blood flow from a retrograde route to regions dominated by the occluded artery, correlates with extensive penumbra and small core infarction [3]. Furthermore, the status of leptomeningeal collaterals can predict the recanalization rate [4], hemorrhagic transformation [5], and outcomes after EVT [6].

Emerging evidence suggests that older age, hypertension, and metabolic syndrome affect the recruitment of leptomeningeal collaterals [7–9]; however, the mechanism remains unclear. In animal models, these risk factors may result in vasodilatory dysfunction of the leptomeningeal collaterals [10]. Given that the leptomeningeal collateral is categorized as microvasculature in anatomy and physiology, investigating the impact of cerebral small vessel disease (CSVD) on it shows a strong rationale.

Leukoaraiosis, or white matter hyperintensity, is a core neuroimaging type of CSVD, and the pathogenesis of its occurrence is relevant to chronic ischemia of white matter caused by luminal stenosis or occlusion of arterioles, a part of microvasculature [11]. In addition, both computed tomography (CT) and magnetic resonance imaging (MRI) can be used as assessment methods for leukoaraiosis [12]. However, other types of CSVD are primarily evaluated by MRI, which is restricted to emergency patients. Therefore, more studies aim to assess the association between leukoaraiosis and leptomeningeal collaterals in patients treated with EVT.

Some studies demonstrated that severe leukoaraiosis was associated with poor recruitment of leptomeningeal collaterals [13–16], and others found that the status of leptomeningeal collateral was not affected by leukoaraiosis [17–22]. No randomized controlled trials have been designed to address this issue. In the present study, we conducted a meta-analysis to explore the effect of leukoaraiosis on leptomeningeal collaterals in acute ischemic stroke treated with EVT.

## Methods

### Search strategy

We performed the meta-analysis following the Preferred Reporting Items for Systematic Reviews and Meta-Analyses (PRISMA) guidelines, 2020 edition [23]. Next, we systematically screened the PubMed, Embase, and Cochrane Library databases from inception to August 2022 by utilizing the terms: cerebral small vessel disease (CSVD), leukoaraiosis, white matter, and collateral. The detailed strategy is shown in Additional file 1.

### Study selection

Followed items were eligible for inclusion criteria: (1) Observational studies; (2) patients with acute ischemic stroke in anterior circulation treated with EVT; (3) exploring the association of CSVD with collateral circulation. The animal experiments, review and meta-analysis, conference abstracts, case reports, and non-English articles were not part of our study.

### Data extraction

After finding and removing the duplicates, two reviewers independently read titles and abstracts and extracted data from full texts based on selection criteria. The following variables were collected: the first author, publication year, country, recruitment time, sample size, age, sex, occlusion position, stroke pathogenesis, and methods of assessment for leukoaraiosis and leptomeningeal collaterals. Severe leukoaraiosis was defined as van Swieten scale (VSS) ≥ 2 scores [15, 17, 20], total Fazekas > 2 [13, 16, 21], as well as deep Fazekas 2 to 3 or periventricular Fazekas 3 [14, 18, 22]. Diagnostic criteria of poor leptomeningeal collaterals included American Society of Intervention and Therapeutic Neuroradiology/Society of Interventional Radiology (ASITN/SIR) scores < 3 [13, 18, 20–22], contrast filling ≤ 50% on the occluded territory [14–16], no collateral filling [19], and less than contralateral hemisphere [17].

### Quality assessment

We utilized the Newcastle–Ottawa Scale (NOS) to evaluate the risk of bias, which scored from selection, comparability, and outcome sections and showed good quality with no fewer than 6 [24].

### Statistical analysis

To explore the association of leukoaraiosis with poor leptomeningeal collaterals, we conducted pooled OR and 95% confidence interval (CI) through the inverse variance method (RevMan 5.3). Heterogeneity was testified by *I*^2^ statistic. When *I*^2^ > 50%, we analyzed data based on a random-effect model, and when *I*^2^ ≤ 50%, we used the fixed-effect model. Furthermore, publication bias was examined by the funnel plot (RevMan 5.3) and quantified through Egger’s test (Stata 14.0). Aiming to elucidate heterogeneity, we incorporated covariates that affect the effect size into meta-regression for continuous variates and subgroup meta-analysis for categorial variates (Stata 14.0). Variates of sample size, mean age, and the number of female patients were delivered for meta-regression. Others were assigned for subgroup analysis, including countries (USA versus France versus China), assessment scales of severe leukoaraiosis (the Fazekas scale versus non-Fazekas scales), imaging methods of leukoaraiosis (CT versus MRI), imaging methods of leptomeningeal collaterals [digital subtraction angiography (DSA) versus CT angiography (CTA)]. The statistical significance of the P value was set at 0.05.

## Results

### Study selection

**Fig. 1:**
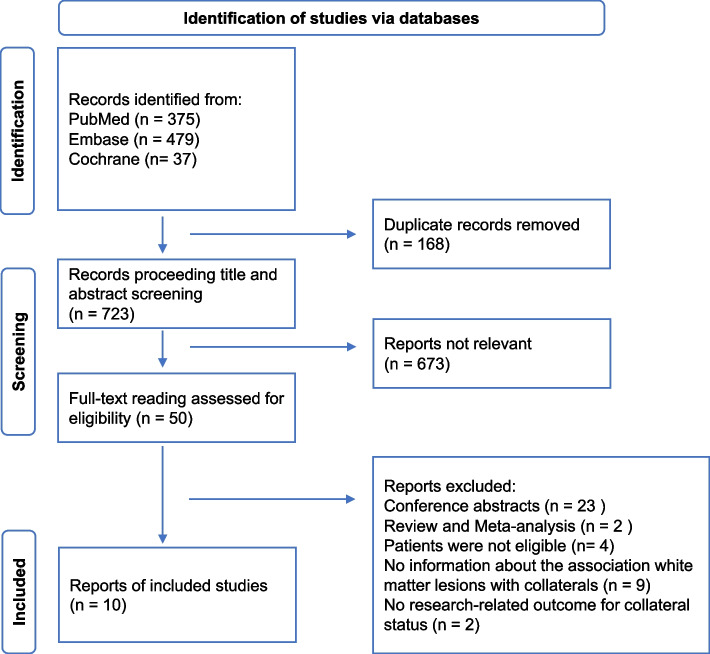
PRISMA screening flowchart

A total of 891 items were retrieved according to the customized strategy. Then, 168 duplicate publications were removed before reviewing the titles and abstracts, of which 50 studies required full-text reading. Last, this study included ten studies [13–22] (Fig. 1).

### Study characteristics

From 2012 through 2022, 1606 patients with acute ischemic stroke in anterior circulation treated with EVT were screened at the ten sites of observational studies from China, the USA, and France. Details on age, number of female patients, occlusion position, stroke pathogenesis, and assessment methods for leukoaraiosis and leptomeningeal collaterals are shown in Table 1. Henninger et al. [17] in 2012 were included in the pooled analysis due to its criteria of patient selection suitable for EVT, evidenced as the first-line therapy for this group in 2018 [1]. All ten studies showed poor leptomeningeal collaterals as an outcome. Consequently, we extracted data from the ten studies to explore the association of leukoaraiosis with poor leptomeningeal collaterals.

**Table 1:** Summary of the included studies

										**Leukoaraiosis**			**Leptomeningeal collaterals**			
**Author** **(Year)**	**Country**	**Recruitment year**	**Sample** **, n**	**Severe leukoaraiosis/** **poor collateral, n**	**Severe leukoaraiosis/** **good collateral, n**	**Age [Mean ± SD or Median (IQR)]**	**Female, n (%)**	**Occlusion** **position**	**Stroke etiology,** **n (%)**	**Image**	**Scale**	**Definition of severe leukoaraiosis**	**Image**	**Scale**	**Definition of** **poor leptomeningeal collaterals**	**NOS**
Henninger (2012) [17]	USA	2007–2010	87	19/68	2/19	67.0 ± 16.0	39 (45.0)	intracranial ICA, M1, M2	LAA 20 (23) CE 38 (44) Others 29 (33)	CT	VSS	≥ 3	CTA	proposed by Lima et al	absent or less than the contralateral hemisphere	4
Eker (2019) [18]	France	2013–2018	240	19/104	33/136	68.7 ± 16.1	118 (49.2)	intracranial ICA, M1, M2	NA	MRI	Fazekas	Deep Fazekas 2–3; Periventricular Fazekas 3	DSA	ASITN/SIR	0–2 score on the occluded territory	6
Lin (2020) [14]	USA	2012–2017	100	24/46	6/54	64.6 ± 16.1	55 (55)	ICA, M1, M2	ICAS 33 (33) TL 32 (32)	MRI	Fazekas	Deep Fazekas 2–3; Periventricular Fazekas 3	CTA	Tan score	contrast filling < 50% on the occluded territory	6
Mark (2020) [13]	USA	NA	178	33/49	48/129	67.6 ± 14.8	91 (51.1)	Anterior circulation	NA	CT	Fazekas	Total Fazekas > 2	CTA/ DSA	ASITN/SIR	0–1 score on the occluded territory	6
Mechtouff (2020) [21]	France	2013–2019	293	NA/33^a^	NA/76^a^	67.1 ± 16.2	133 (45.6)	intracranial ICA, M1	NA	MRI	Fazekas	Total Fazekas > 2	DSA	ASITN/SIR	0–2 score on the occluded territory	4
Mutzenbach (2020) [19]	USA	2012–2019	209	9/50	26/159	75(63–81)	111 (53.1)	M1	LAA 23 (11) CE 115(55)	MRI/ CT	ARWMC	Top 25 percentiles	CTA	Tan score	contrast filling = 0 on the occluded territory	4
Mikat (2020) [20]	USA	2012–2016	144	7/38	15/106	68(57–81)	71 (49.3)	Anterior circulation	LAA 30 (21) CE 74 (51) ESUS 27(19)	CT	VSS	≥ 3	DSA	ASITN/SIR	0–2 score on the occluded territory	4
Forestier (2022) [22]	France	2015–2020	312	59/207	27/105	67.8 ± 14.9	146 (46.8)	intracranial ICA or M1	NA	MRI	Fazekas	Deep Fazekas 2–3; Periventricular Fazekas 3	DSA	ASITN/SIR	0–2 score on the occluded territory	6
Hashimoto (2022) [16]	USA	2015–2019	108	36/41	33/67	76.5 (63.3–86.0)	49 (45.3)	terminus ICA, M1, M2	CE 82 (76) TL 12 (11) ESUS 10 (9)	MRI	Fazekas	Total Fazekas > 2	CTA	Tan score	contrast filling ≤ 50% on the occluded territory	6
Zhou (2022) [15]	China	2018–2021	119	16/40	15/79	74(61–84)	51 (42.9)	M1, M2	ICAS 93 (78.2)	MRI	VSS	≥ 2	CTA	Tan score	contrast filling < 50% on the occluded territory	6

*LAA* Large-artery atherosclerosis, *CE* Cardiogenic embolism, *TL* Tandem lesions, *ICAS* Intracranial atherosclerosis, *ESUS* Embolic stroke of undetermined source, *ICA* Internal carotid artery, *M1* The first segment of the middle cerebral artery, *NOS* Newcastle–Ottawa Scale, *VSS* Van Swieten scale, *ASITN/SIR* American Society of Intervention and Therapeutic Neuroradiology/Society of Interventional Radiology, *DSA* Digital subtraction angiography, *CTA* Computed tomography angiography, *SD* Standard deviation, *IQR* interquartile range, *NA* Not available

^a^Mechtouff et al. reported odds ratio (OR) about the association of severe leukoaraiosis with poor leptomeningeal collateral based on 109 patients who acquired the scores of leptomeningeal collaterals

### Quality assessment

Six studies were demonstrated for good quality with NOS ≥ 6 (Table 1), and specific items were found in Additional file 2.

### Meta-analysis outcomes

**Fig. 2:**
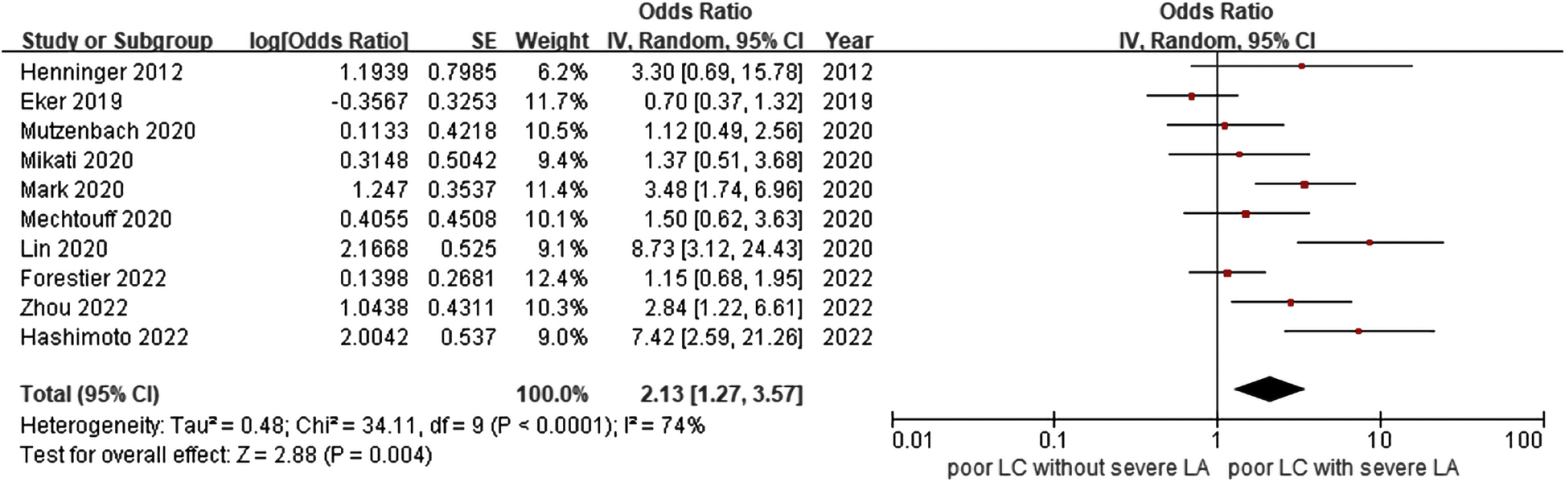
Forest map of the association between leukoaraiosis and poor leptomeningeal collaterals

**Fig. 3:**
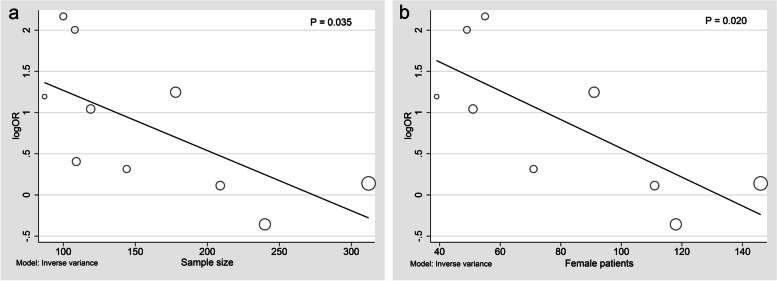
Bubble plot of the association of sample size and female patients with effect size. **a** the association between sample size and effect size; (**b)** the association between number of female patients and effect size

**Fig. 4:**
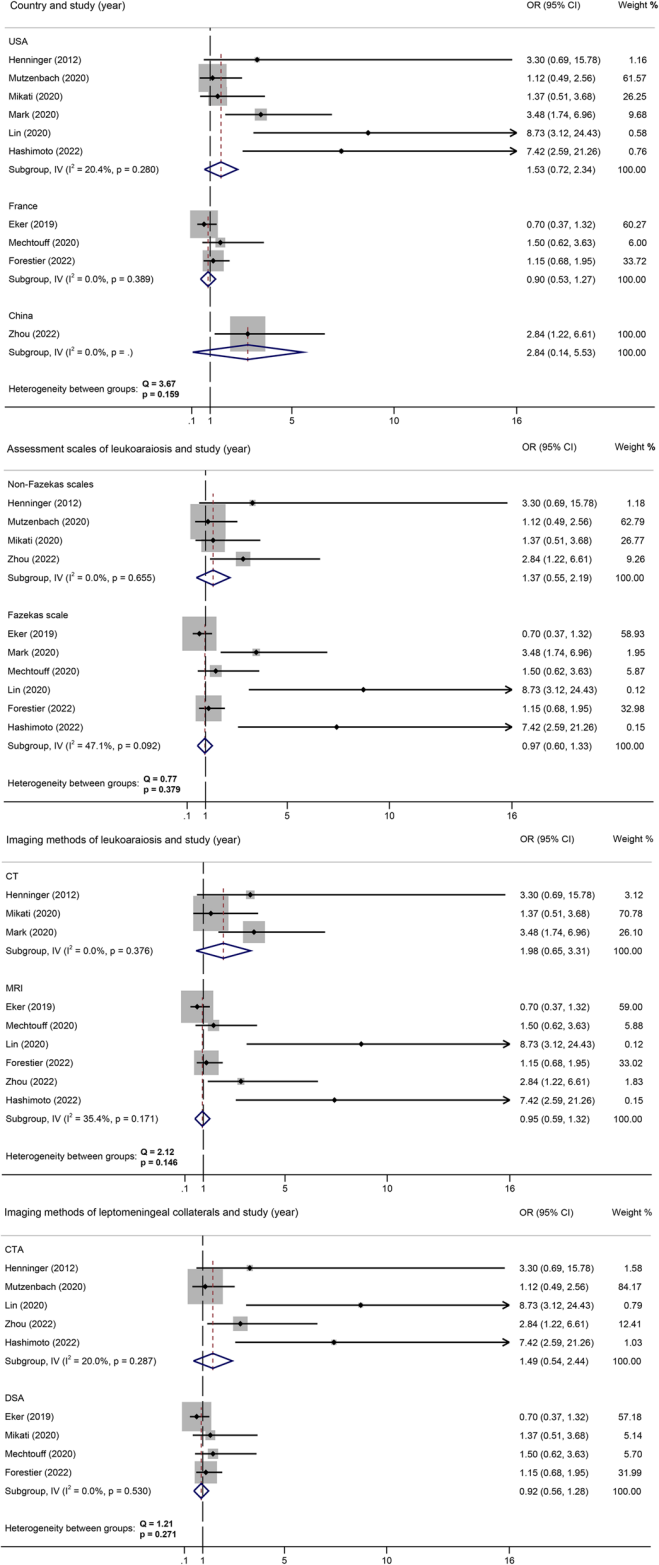
Subgroups meta-analysis of the association between leukoaraiosis and poor leptomeningeal collaterals. Legend: MRI, magnetic resonance image; CT, computed tomography; DSA, digital subtraction angiography; OR, odds ratio. Mutzenbach et al. reported both CT and MRI as images of leukoaraiosis; therefore, this study was excluded from analysis in this subgroup. Mark et al. reported both DSA and CTA as images of leptomeningeal collateral; therefore, this study was excluded from analysis in this subgroup

The meta-analysis, utilizing a random-effect model with *I*^2^ of 74%, showed a significant correlation between severe leukoaraiosis and poor leptomeningeal collaterals (pooled OR 2.13, 95% CI 1.27–3.57, *P* = 0.004) (Fig. 2). Meta-regression indicated that sample size (coefficient = -0.007299, *P* = 0.035) and the number of female patients (coefficient = -0.0174709, *P* = 0.020) rather than mean age (*P* = 0.991) were potential sources of heterogeneity. As Fig. 3 shown, the effect size decreased with increasing sample size (Fig. 3a) and the number of female patients (Fig. 3b). Mechtouff et al. [21] did not report the number of females out of 109 patients as an outcome analysis; therefore, the covariate of this study was eliminated from meta-regression (Fig. 3b). Among categorical variates of countries (Q = 3.67, *P* = 0.159), assessment scales of leukoaraiosis (Q = 0.77, *P* = 0.379), images of leukoaraiosis (Q = 2.12, *P* = 0.146), images of leptomeningeal collaterals (Q = 1.21, *P* = 0.271), all the subgroup analysis demonstrated no significant difference between groups (Fig. 4).

### Publication bias

**Fig. 5:**
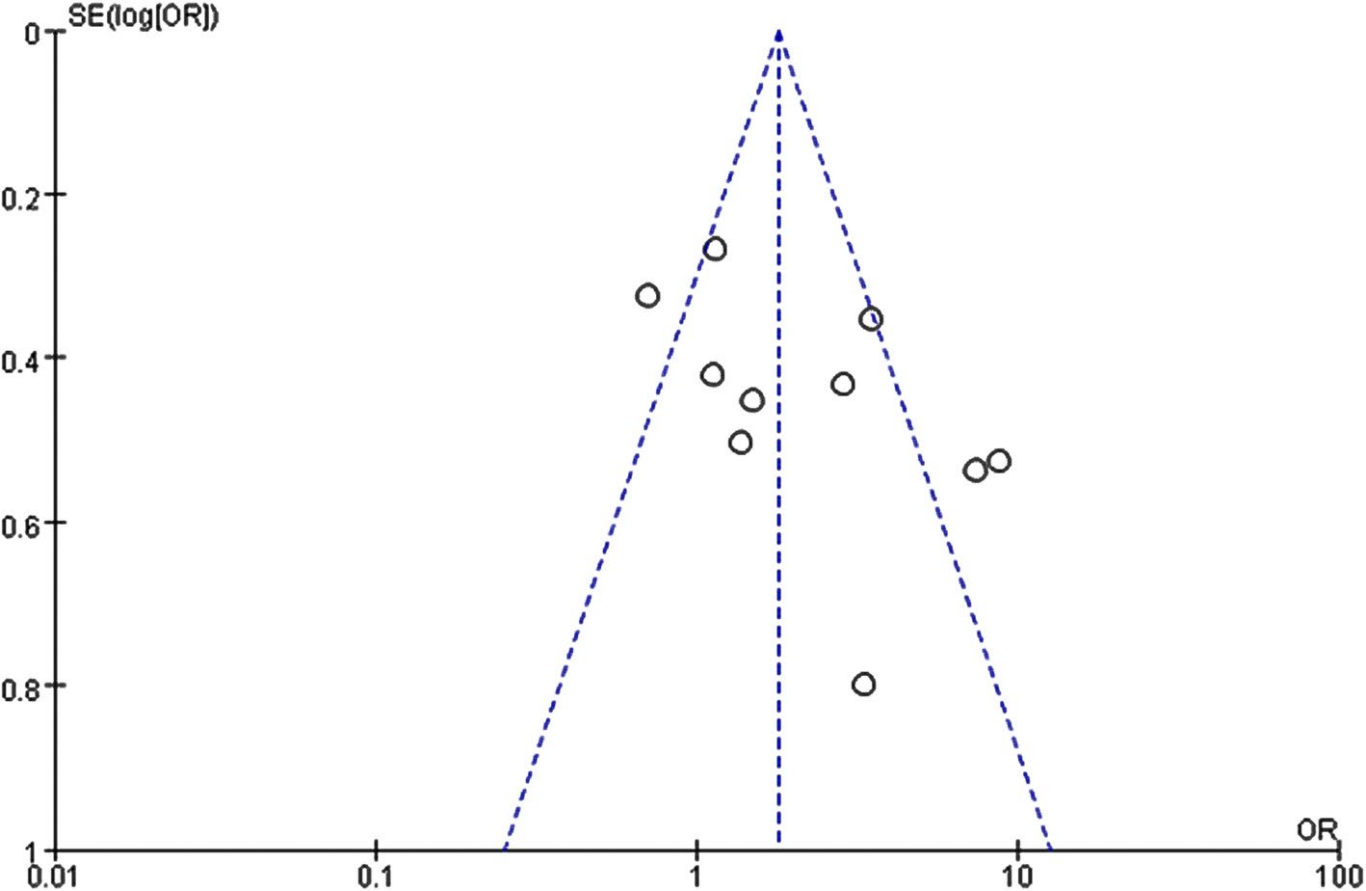
Funnel plot of the association between leukoaraiosis and poor leptomeningeal collaterals

The funnel plot showed that three studies were away from the interval range of effect value (Fig. 5). Egger’s test (*p* = 0.085) showed no significant publication bias for included studies.

## Discussion

In this study, we found that severe leukoaraiosis was associated with poor recruitment of leptomeningeal collaterals in patients treated with EVT. Compared with non-severe leukoaraiosis, severe leukoaraiosis increased 2.13 times risk for emerging poor leptomeningeal collaterals.

Included studies showed no publication bias; nevertheless, our results demonstrated that the sample size and the number of female patients correlated with significant heterogeneity based on the meta-regression and subgroup analysis. Heterogeneity in sample size may be due to the fact that larger sample size is usually associated with greater statistical power, a smaller source of error, and higher study quality. Heterogeneity caused by sex may be related to Estrogen, a sex steroid, which showed protection for the neurovascular unit, including the leptomeningeal collaterals and brain parenchyma [25, 26]. Female patients in our study were in the stage of menopause; therefore, low-concentration Estrogen may show poor neuroprotection.

According to our report, the study countries among China, USA, and France showed no heterogeneity in concluding the association of severe leukoaraiosis with poor leptomeningeal collaterals. However, a recent meta-analysis demonstrated that patients from the USA contributed to the heterogeneity [27]. This discrepancy may result from two categories (USA vs. Non-USA) for the previous meta-analysis and three for ours. In addition, all patients in our study were suitable for EVT due to large vessel occlusion, while part of the formers was included with large vessel stenosis. Types of stroke etiology vary among different countries; for example, intracranial atherosclerosis (ICAS) is a common mechanism in China [28], and cardiogenic embolism (CE) in Western counties [29]. Patients with atherosclerosis-related stroke, including intracranial or extracranial segment, constructed better collateral flow to resist chronic ischemia compared with CE-related sudden occlusion [30, 31]. However, from the perspective of study design for stroke etiology, only Hashimoto et al. demonstrated that severe leukoaraiosis decreased the recruitment of leptomeningeal collaterals in CE-related occlusion [16]. In our study, we could not conduct subgroup analysis based on the stroke subtype due to the absence of stratification in original studies. Further research is required to address this issue.

Accurate evaluation of leukoaraiosis was essential to predict the status of leptomeningeal collaterals. Our results found no heterogeneity among visual assessment scales and imaging methods. To date, the Fazekas devised in 1987 [32], VSS in 1990 [33], and age-related white matter change (ARWMC) in 2001 [12] were still the most common scales to assess leukoaraiosis and any of the scales showed good efficiency between inter- and intra-raters. MRI showed more sensitivity than CT to detect white matter changes, especially for small lesions, whereas severe lesions were evaluated equally with CT and MRI [12, 34]. Although volumetric quantification of leukoaraiosis was superior to rating scales [35], it may be poorly applicable to clinical practice. The selection of patients treated before EVT demands assessment methods compatible with brevity and effectiveness to shorten recanalization time. Hence, to predict the status of leptomeningeal collaterals, we may choose CT as a priority to evaluate whether the patients treated with EVT emerge with severe leukoaraiosis. Similarly, our results found no heterogeneity between the imaging techniques used to assess leptomeningeal collaterals (CTA and DSA). However, the dichotomous leptomeningeal collaterals for ordinal classification in the original literature were not always consistent, so the classification systems for leptomeningeal collaterals may be a potential source of heterogeneity.

Arteriolosclerosis is a common pathogenic classification of leukoaraiosis and belongs to age-related and vascular risk-factor-related small vessel disease [36]. Age and heredity are non-interventional factors; however, decreasing the variability of blood pressure may alleviate the process of leukoaraiosis [37]. Furthermore, proper management of diabetes and ceasing smoking show neuroprotection for white matter [38, 39]. Recently, Dl-3-butyl phthalide, a neuroprotective drug approved in clinical practice for the Chinese in 2005, established its value in improving cerebral hypoperfusion by increasing the flow of collateral circulation in patients with carotid artery atherosclerotic stenosis [40]. However, the protective effect of Dl-3-butyl phthalide on the white matter was just demonstrated in mice models [41]. This drug might be a promising therapy for leukoaraiosis in clinical practice.

The strengths of our study include one study population (patients treated with EVT) and a detailed exploration of heterogeneity by meta-regression and subgroup analysis. Cautiously, our results are appropriate for patients treated with EVT. We excluded non-English studies, which may result in a selection bias due to published language. The relatively small number of original studies weakens the ability to draw meaningful conclusions about subgroups. More research is needed to validate our findings.

## Conclusion

In summary, severe leukoaraiosis was associated with poor leptomeningeal collaterals in patients treated with EVT. Further studies may focus on whether the finding applies to different stroke subtypes.

## Supplementary Information

**Additional file 1: Table 1. **A search strategy in the PubMed database.

**Additional file 2: Table 2. **Quality assessment of the included studies using the Newcastle-Ottawa scale.

## Data Availability

The datasets supporting the conclusions of this article are included within the paper and its additional files.

## Abbreviations

EVT: Endovascular therapy
CSVD: Cerebral small vessel disease
CT: Computed tomography
MRI: Magnetic resonance imaging
CI: Confidence interval
ICAS: Intracranial atherosclerosis
CE: Cardiogenic embolism

## References

[1] Powers WJ, Rabinstein AA, Ackerson T, Adeoye OM, Bambakidis NC, Becker K (2019). Guidelines for the early management of patients with acute ischemic stroke: 2019 update to the 2018 guidelines for the early management of acute ischemic stroke: a guideline for healthcare professionals from the American Heart Association/American Stroke Association. Stroke.

[2] Warach SJ, Luby M, Albers GW, Bammer R, Bivard A, Campbell BC (2016). Acute stroke imaging research roadmap iii imaging selection and outcomes in acute stroke reperfusion clinical trials: consensus recommendations and further research priorities. Stroke.

[3] Miteff F, Levi CR, Bateman GA, Spratt N, McElduff P, Parsons MW (2009). The independent predictive utility of computed tomography angiographic collateral status in acute ischaemic stroke. Brain.

[4] Garcia-Tornel A, Ciolli L, Rubiera M, Requena M, Muchada M, Pagola J (2021). Leptomeningeal Collateral Flow Modifies Endovascular Treatment Efficacy on Large-Vessel Occlusion Strokes. Stroke.

[5] Regenhardt RW, Gonzalez RG, He J, Lev MH, Singhal AB (2022). Symmetric CTA Collaterals Identify Patients with Slow-progressing Stroke Likely to Benefit from Late Thrombectomy. Radiology.

[6] Roman LS, Menon BK, Blasco J, Hernandez-Perez M, Davalos A, Majoie C (2018). Imaging features and safety and efficacy of endovascular stroke treatment: a meta-analysis of individual patient-level data. Lancet Neurol.

[7] Wiegers EJA, Mulder M, Jansen IGH, Venema E, Compagne KCJ, Berkhemer OA (2020). Clinical and Imaging Determinants of Collateral Status in Patients With Acute Ischemic Stroke in MR CLEAN Trial and Registry. Stroke.

[8] Nannoni S, Sirimarco G, Cereda CW, Lambrou D, Strambo D, Eskandari A (2019). Determining factors of better leptomeningeal collaterals: a study of 857 consecutive acute ischemic stroke patients. J Neurol.

[9] Fujita K, Tanaka K, Yamagami H, Ide T, Ishiyama H, Sonoda K (2019). Detrimental effect of chronic hypertension on leptomeningeal collateral flow in acute ischemic stroke. Stroke.

[10] Chan SL, Sweet JG, Bishop N, Cipolla MJ (2016). Pial collateral reactivity during hypertension and aging: understanding the function of collaterals for stroke therapy. Stroke.

[11] Fernando MS, Simpson JE, Matthews F, Brayne C, Lewis CE, Barber R (2006). White matter lesions in an unselected cohort of the elderly: molecular pathology suggests origin from chronic hypoperfusion injury. Stroke.

[12] Wahlund LO, Barkhof F, Fazekas F, Bronge L, Augustin M, Sjogren M (2001). A new rating scale for age-related white matter changes applicable to MRI and CT. Stroke.

[13] Mark I, Seyedsaadat SM, Benson JC, Kallmes DF, Rabinstein AA, Brinjikji W (2020). Leukoaraiosis and collateral blood flow in stroke patients with anterior circulation large vessel occlusion. J Neurointerv Surg.

[14] Lin MP, Brott TG, Liebeskind DS, Meschia JF, Sam K, Gottesman RF (2020). Collateral recruitment is impaired by cerebral small vessel disease. Stroke.

[15] Zhou JY, Shi YB, Xia C, Lu CQ, Tang TY, Lu T (2022). Beyond collaterals: brain frailty additionally improves prediction of clinical outcome in acute ischemic stroke. Eur Radiol.

[16] Hashimoto T, Kunieda T, Honda T, Scalzo F, Ali L, Hinman JD (2022). Reduced Leukoaraiosis, Noncardiac Embolic Stroke Etiology, and Shorter Thrombus Length Indicate Good Leptomeningeal Collateral Flow in Embolic Large-Vessel Occlusion. AJNR Am J Neuroradiol.

[17] Henninger N, Lin E, Baker SP, Wakhloo AK, Takhtani D, Moonis M (2012). Leukoaraiosis predicts poor 90-day outcome after acute large cerebral artery occlusion. Cerebrovasc Dis.

[18] Eker OF, Rascle L, Cho TH, Mechtouff L, Derex L, Ong E (2019). Does small vessel disease burden impact collateral circulation in ischemic stroke treated by mechanical thrombectomy?. Stroke.

[19] Mutzenbach JS, Muller-Thies-Broussalis E, Killer-Oberpfalzer M, Griessenauer CJ, Hecker C, Moscote-Salazar LR (2020). severe leukoaraiosis is associated with poor outcome after successful recanalization of M1 middle cerebral artery occlusion strokes. Cerebrovasc Dis.

[20] Mikati AG, Mandelbaum M, Sapnar S, Puri AS, Silver B, Goddeau RP (2020). Impact of leukoaraiosis severity on the association of time to successful reperfusion with 90-day functional outcome after large vessel occlusion stroke. Transl Stroke Res.

[21] Mechtouff L, Nighoghossian N, Amaz C, Buisson M, Berthezène Y, Derex L (2020). White matter burden does not influence the outcome of mechanical thrombectomy. J Neurol.

[22] Forestier G, Agbonon R, Bricout N, Benhassen W, Turc G, Bretzner M (2022). Small vessel disease and collaterals in ischemic stroke patients treated with thrombectomy. J Neurol.

[23] Page MJ, McKenzie JE, Bossuyt PM, Boutron I, Hoffmann TC, Mulrow CD (2021). The PRISMA 2020 statement: an updated guideline for reporting systematic reviews. BMJ.

[24] Wells G, Shea B, O’Connell D, Peterson J, Welch V, Losos M, et al. The Newcaste Ottawa Scale (NOS) for assessing the quality of nonrandomized studies in meta-analyses. Available online from: http://www.ohri.ca/programs/clinical_epidemiology/oxford.asp. Accessed on 30 August 2022.

[25] Raz L (2014). Estrogen and cerebrovascular regulation in menopause. Mol Cell Endocrinol.

[26] Hawkins BT, Davis TP (2005). The blood-brain barrier/neurovascular unit in health and disease. Pharmacol Rev.

[27] Xu M, Guo W, Rascle L, Mechtouff L, Nighoghossian N, Eker O (2022). Leukoaraiosis distribution and cerebral collaterals: a systematic review and meta-analysis. Front Neurol.

[28] Wang Y, Zhao X, Liu L, Soo YO, Pu Y, Pan Y (2014). Prevalence and outcomes of symptomatic intracranial large artery stenoses and occlusions in China: the Chinese Intracranial Atherosclerosis (CICAS) Study. Stroke.

[29] Goyal M, Menon BK, van Zwam WH, Dippel DW, Mitchell PJ, Demchuk AM (2016). Endovascular thrombectomy after large-vessel ischaemic stroke: a meta-analysis of individual patient data from five randomised trials. Lancet.

[30] Rebello LC, Bouslama M, Haussen DC, Grossberg JA, Dehkharghani S, Anderson A (2017). Stroke etiology and collaterals: atheroembolic strokes have greater collateral recruitment than cardioembolic strokes. Eur J Neurol.

[31] Hassler E, Kneihsl M, Deutschmann H, Hinteregger N, Magyar M, Wiesspeiner U (2020). Relationship between stroke etiology and collateral status in anterior circulation large vessel occlusion. J Neurol.

[32] Fazekas F, Chawluk JB, Alavi A, Hurtig HI, Zimmerman  RA (1987). MR signal abnormalities at 1.5 T in Alzheimer's dementia and normal aging. AJR Am J Roentgenol.

[33] van Swieten JC, Hijdra A, Koudstaal PJ, van Gijn J (1990). Grading white matter lesions on CT and MRI: a simple scale. J Neurol Neurosurg Psychiatry.

[34] Fazekas F, Barkhof F, Wahlund LO, Pantoni L, Erkinjuntti T, Scheltens P (2002). CT and MRI rating of white matter lesions. Cerebrovasc Dis.

[35] Kapeller P, Barber R, Vermeulen RJ, Ader H, Scheltens P, Freidl W (2003). Visual rating of age-related white matter changes on magnetic resonance imaging: scale comparison, interrater agreement, and correlations with quantitative measurements. Stroke.

[36] Pantoni L (2010). Cerebral small vessel disease: from pathogenesis and clinical characteristics to therapeutic challenges. Lancet Neurol.

[37] Ma Y, Song A, Viswanathan A, Blacker D, Vernooij MW, Hofman A (2020). Blood Pressure Variability and Cerebral Small Vessel Disease: A Systematic Review and Meta-Analysis of Population-Based Cohorts. Stroke.

[38] van Harten B, Oosterman JM, Potter van Loon BJ, Scheltens P, Weinstein  HC (2007). Brain lesions on MRI in elderly patients with type 2 diabetes mellitus. Eur Neurol.

[39] Arntz RM, van den Broek SM, van Uden IW, Ghafoorian M, Platel B, Rutten-Jacobs LC (2016). Accelerated development of cerebral small vessel disease in young stroke patients. Neurology.

[40] Chen D, Yin Y, Shi J, Yang F, Wang K, Zhao F (2020). DL-3-n-butylphthalide improves cerebral hypoperfusion in patients with large cerebral atherosclerotic stenosis: a single-center, randomized, double-blind, placebo-controlled study. BMC Neurol.

[41] Han QY, Zhang H, Zhang X, He DS, Wang SW, Cao X (2019). dl-3-n-butylphthalide preserves white matter integrity and alleviates cognitive impairment in mice with chronic cerebral hypoperfusion. CNS Neurosci Ther.

