# Supplementary material for: Effect of leukoaraiosis on collateral circulation in acute ischemic stroke treated with endovascular therapy: a meta-analysis
Source: BMC Neurol. 2023 Jun 1;23:212. doi: 10.1186/s12883-023-03266-8 (PMC10233903; doi:10.1186/s12883-023-03266-8)
Supplement: Supplementary file 1 — Additional file 1: Table 1. A search strategy in the PubMed database. [file 12883_2023_3266_MOESM1_ESM.docx]

**Additional Table1.** A search strategy in the PubMed database

| Steps | Queries | Items of studies |
| --- | --- | --- |
| #1 | Cerebral Small Vessel Diseases [Mesh] | 9053 |
| #2 | cerebral small vessel disease | 12087 |
| #3 | Leukoaraiosis [Mesh] | 766 |
| #4 | leukoaraiosis | 766 |
| #5 | white matte | 70475 |
| #6 | collateral | 51268 |
| #7 | (#1 or #2 or #3 or #4 or #5) and #6 | 375 |
