# Supplementary material for: Effect of leukoaraiosis on collateral circulation in acute ischemic stroke treated with endovascular therapy: a meta-analysis
Source: BMC Neurol. 2023 Jun 1;23:212. doi: 10.1186/s12883-023-03266-8 (PMC10233903; doi:10.1186/s12883-023-03266-8)
Supplement: Supplementary file 2 — Additional file 2: Table 2. Quality assessment of the included studies using the Newcastle-Ottawa scale. [file 12883_2023_3266_MOESM2_ESM.docx]

**Additional Table 2.** Quality assessment of the included studies using Newcastle-Ottawa scale

| **Study** | **Selection** | | | | **Comparability** | **Outcome** | | | **Score** |
| --- | --- | --- | --- | --- | --- | --- | --- | --- | --- |
|  | 1) | 2) | 3) | 4) | 1) | 1) | 2) | 3) |  |
| Henninger (2012) | * | * |  |  |  | * | * |  | 4 |
| Eker (2019) | * | * |  |  | ** | * | * |  | 6 |
| Lin (2020) | * | * |  |  | ** | * | * |  | 6 |
| Mark (2020) | * | * |  |  | ** | * | * |  | 6 |
| Mechtouff (2020) | * | * |  |  |  | * | * |  | 4 |
| Mutzenbach (2020) | * | * |  |  |  | * | * |  | 4 |
| Mikati (2020) | * | * |  |  |  | * | * |  | 4 |
| Forestier (2022) | * | * |  |  | ** | * | * |  | 6 |
| Hashimoto (2022) | * | * |  |  | ** | * | * |  | 6 |
| Zhou (2022) | * | * |  |  | ** | * | * |  | 6 |

Selection: 1) Is the case definition adequate? 2) Representativeness of the cases; 3) Selection of Controls; 4) Definition of Controls.

Comparability: Comparability of cases and controls on the basis of the design or analysis Comparability.

Outcome: 1) Ascertainment of exposure; 2) Same method of ascertainment for cases and controls; 3) Non response rate.
